# Production of Structurally Defined Chito-Oligosaccharides with a Single *N*-Acetylation at Their Reducing End Using a Newly Discovered Chitinase from *Paenibacillus pabuli*

**DOI:** 10.1021/acs.jafc.0c06804

**Published:** 2021-03-10

**Authors:** Jing Li, Damao Wang, Shu-Chieh Chang, Pi-Hui Liang, Vaibhav Srivastava, Shih-Yun Guu, Jiun-Jie Shie, Kay-Hooi Khoo, Vincent Bulone, Yves S. Y. Hsieh

**Affiliations:** † College of Life Sciences, Shanghai Normal University, Shanghai 220234, PR China; ‡ Division of Glycoscience, Department of Chemistry, School of Engineering Sciences in Chemistry, Biotechnology and Health, Royal Institute of Technology (KTH), AlbaNova University Center, Stockholm SE10691, Sweden; § School of Pharmacy, College of Pharmacy, Taipei Medical University, 250 Wuxing Street, Taipei 110, Taiwan; ∥ College of Food Science, Southwest University, Chongqing 400715, PR China; ⊥ School of Pharmacy, College of Medicine, National Taiwan University, Taipei 100, Taiwan; # Institute of Biological Chemistry, Academia Sinica, 128 Academia Road Sec. 2, Nankang, Taipei 115, Taiwan; ¶ Institute of Chemistry, Academia Sinica, 128 Academia Road Sec. 2, Nankang, Taipei 115, Taiwan; ∇ School of Agriculture, Food and Wine, The University of Adelaide, Urrbrae 5064, Australia; ○ Genomics Research Center, Academia Sinica, 128 Academia Road Sec. 2, Nankang, Taipei 115, Taiwan

**Keywords:** Chitinase, Chito-oligosaccharides, Chitin, Chitosan

## Abstract

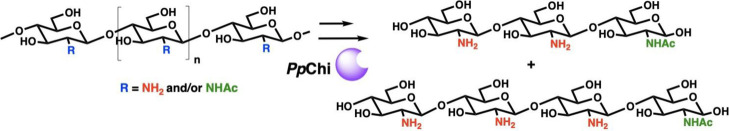

Partially acetylated chito-oligosaccharides (paCOSs) are bioactive compounds with potential medical applications. Their biological activities are largely dependent on their structural properties, in particular their degree of polymerization (DP) and the position of the acetyl groups along the glycan chain. The production of structurally defined paCOSs in a purified form is highly desirable to better understand the structure/bioactivity relationship of these oligosaccharides. Here, we describe a newly discovered chitinase from *Paenibacillus pabuli* (*Pp*Chi) and demonstrate by mass spectrometry that it essentially produces paCOSs with a DP of three and four that carry a single *N-*acetylation at their reducing end. We propose that this specific composition of glucosamine (GlcN) and *N*-acetylglucosamine (GlcNAc) residues, as in GlcN_(*n*)_GlcNAc_1_, is due to a subsite specificity toward GlcN residues at the −2, −3, and −4 positions of the partially acetylated chitosan substrates. In addition, the enzyme is stable, as evidenced by its long shelf life, and active over a large temperature range, which is of high interest for potential use in industrial processes. It exhibits a *k*_cat_ of 67.2 s^–1^ on partially acetylated chitosan substrates. When *Pp*Chi was used in combination with a recently discovered fungal auxilary activity (AA11) oxidase, a sixfold increase in the release of oligosaccharides from the lobster shell was measured. *Pp*Chi represents an attractive biocatalyst for the green production of highly valuable paCOSs with a well-defined structure and the expansion of the relatively small library of chito-oligosaccharides currently available.

## Introduction

Chitin is a natural polymer that is typically prepared from the exoskeleton of crustaceans and mushroom cell walls. Together with cellulose, it represents one of the most abundant biopolymers on earth.^1^ Chitin is biodegradable and biocompatible, and its deacetylated form chitosan exhibits antimicrobial properties, which can be exploited in a large range of applications,^2^ for example, in food packaging materials,^3^ food additives,^4^ medical consumables,^5^ and crop-protecting formulations against pathogenic microorganisms.^6,7^ However, chitin is not soluble at a neutral pH, which limits its use in more advanced biotechnological applications.^8^ Oligosaccharides that have increased solubility can be derived from chitin upon acid or enzymatic hydrolysis. They are composed of *N*-acetylglucosamine (GlcNAc) and glucosamine (GlcN) residues and are referred to as partially acetylated chito-oligosaccharides (paCOSs). Many reports have suggested that paCOSs are potent biologics with potential medical applications based on their activities, such as wound-healing materials,^9^ vectors in gene therapy,^10^ tissue repair,^11^ reduction of cancer metastasis,^12,13^ and anti-fungal and antimalarial formulations.^14,15^

**Scheme 1:**
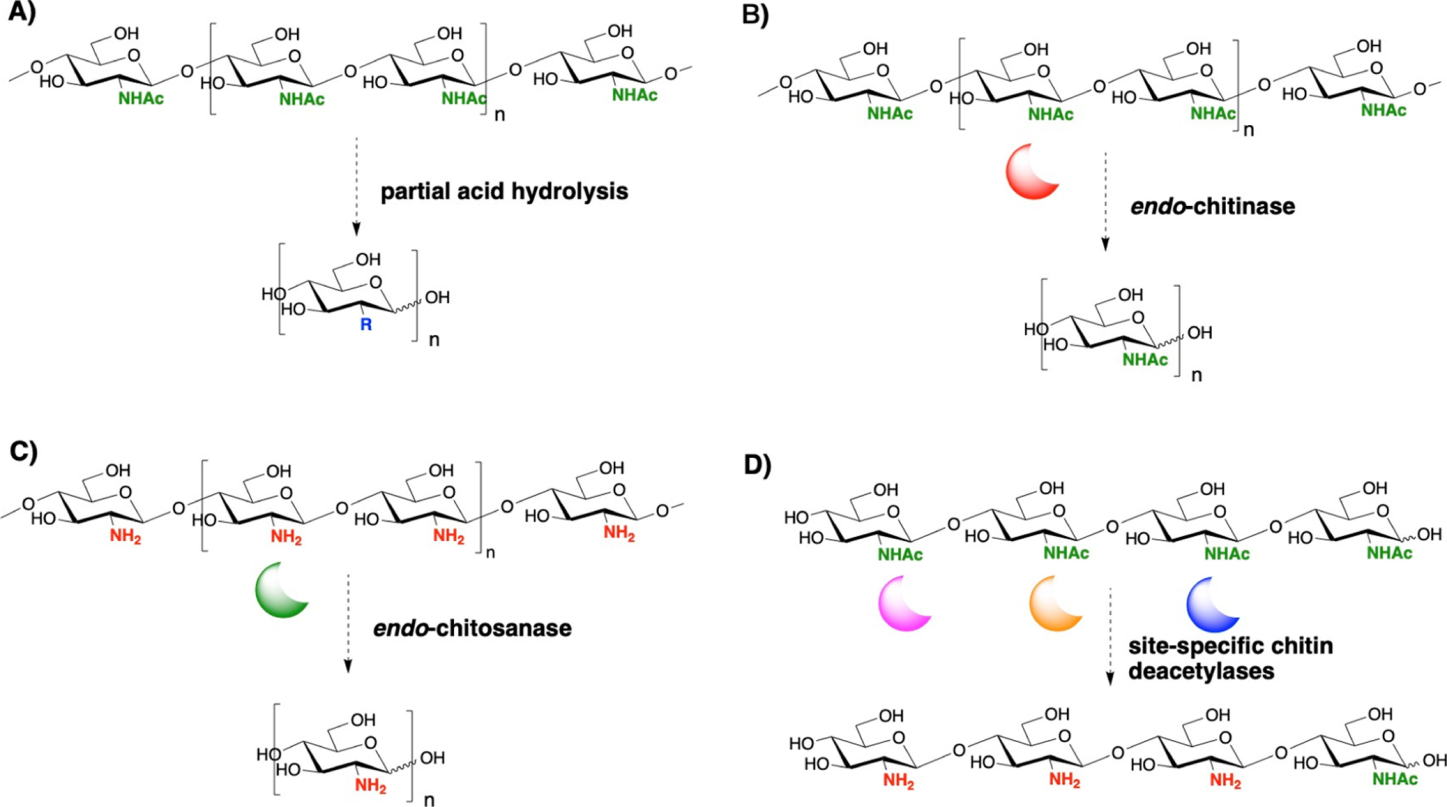
Chemical and Enzymatic Routes for the Production of Chito-Oligosaccharides (COSs): (A) Partial Acid Hydrolysis Degrades Chitin from Crustacean Shells or Squid Pens Into Mixtures of GlcN or GlcNAc Residues, paCOSs, And/or Fully Deacetylated Oligosaccharides with Various DPs Depending on the Acid Treatment Conditions (R = NH_2_ or NHAc); (B) *Endo*-Chitinase Hydrolysis of Chitin Typically Generates a Range of COSs with Varying DPs (*n* = 1–9) Depending on the Enzyme and Substrate Used As Well As the Duration of the Treatment; (C) *Endo*-Chitosanase Hydrolysis of fully Deacetylated Chitosan, for example Chitosanase from *Streptomyces griseus*, Leads to the Formation of COSs of Different DPs (*n* = 2–6); and (D) Regioselective Deacetylation Using Site-Specific Chitin Deacetylases to Obtain Homogeneous Glycoform of Partially Deacetylated COSs

Biological activities of paCOSs are often evaluated using mixtures of oligosaccharides that vary in chain length and *N*-acetylation patterns.^1^ How the distribution of acetyl groups along the glycan chains influences the function of paCOSs has been investigated only in a limited number of studies.^16−19^ To address this question in more detail, access to pure paCOSs in single glycoforms with a well-defined acetylation pattern is needed. Currently, the commercial production of chitin oligomers typically employs strong acid treatments at elevated temperatures to initiate the breakdown of the chitin β-1,4 glycosidic linkages.^20−22^ This shortens the production time but results in heterogeneous mixtures of GlcNAc and oligomers. The subsequent use of acid-catalyzed *N*-deacetylation generates paCOSs and fully deacetylated chitosan oligomers^23^ (Scheme 1A). Alternatively, mild acid treatment can also produce paCOSs, but batch quality consistency is difficult to control, and the reaction often concomitantly generates secondary products such as 4-oxopentanoic acid (levulinic acid) or 2,5-anhydro-d-mannose, which are difficult to remove in subsequent purification steps.^24^ Acid treatment is also plagued with environmental concerns, so exploring eco-friendly chitolytic enzyme treatments has become a preferred approach.^25−30^

The chemoenzymatic synthesis of single glycoforms of paCOSs has been achieved by the regioselective removal of acetate from chitin oligosaccharides using chitin deacetylases^31^ (Scheme 1D). However, the method is not straightforward, and the deacetylases currently available are not comprehensive in deploying all possible types of regioselectivity.^32^ Success of this approach is also dependent on having access to pure oligosaccharides as starting materials for the removal of the acetyl groups. Furthermore, optimization is required, for example, to remove unreacted starting oligosaccharides, and the expression of the chitin deacetylases in *Escherichia coli* is typically difficult and accompanied by low yields.^31^ Theoretically, the best and simplest approach to generate paCOSs as single glycoforms is to employ a single chitinase enzyme specific for a defined pattern of acetylation.

**Scheme 2:**
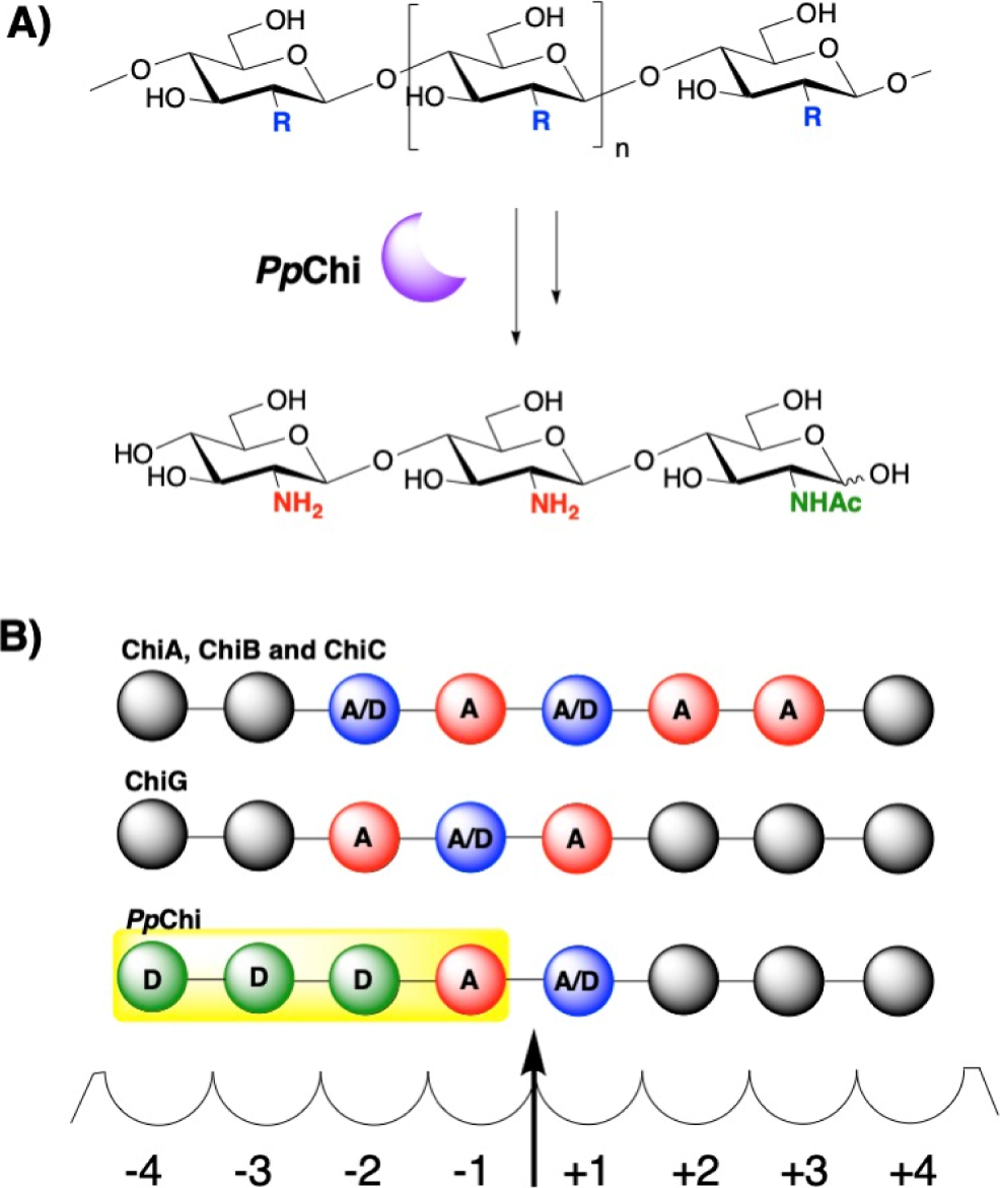
(A) Production of paCOSs with a Defined *N*-Acetylation Pattern (DDA and DDDA) Using *Pp*Chi (R = NH_2_ or NHAc). (B) Subsite Binding and Catalysis at the Active Sites of Various Chitinases, including ChiA, ChiB, ChiC, and ChiG, and Likely Subsite Binding of *Pp*Chi. The Arrow Indicates the Glycosidic Linkage Hydrolyzed

Chitinases act as molecular scissors to hydrolyze chitin into lower molecular weight chito-oligosaccharides. Specifically, there are three common chitinase classes, that is, chitinase A (ChiA), chitinase B (ChiB), and chitinase C (ChiC) in Glycoside Hydrolase (GH) family 18, which all have a substrate-binding site that requires a GlcNAc residue (A) at the −1 subsite position and either an A or GlcN (D) residue at the +1 position (Scheme 2B).^1^ In most cases, these chitinases primarily generate oligomers with GlcNAc-β-1,4-GlcNAc_R_ (AA) motifs at the reducing end, such as the disaccharide GlcNAc-β-1,4-GlcNAc (AA) and the trisaccharide GlcNAc-β-1,4-GlcNAc-β-1,4-GlcNAc_R_ (AAA) (Scheme 1B). In comparison, ChiG from GH family 19 has a unique subsite preference as it liberates the disaccharides AD/AA and the trisaccharide AAD as the dominant products.^33^ The advantage of using chitinases is that these enzymes exhibit high specificity at their subsites, which allows the control of the *N*-acetylation pattern of the final product and the generation of oligomers with more defined DPs. In addition, chitinase reactions are carried out in aqueous buffers, which makes the reactions easier to control and more friendly to the environment.

Here, we report the use of a newly discovered chitinase from *Paenibacillus pabuli* (*Pp*Chi) that liberates two distinctive oligomers in high abundance, that is, the trisaccharide GlcN-β-1,4-GlcN-β-1,4-GlcNAc_R_ (DDA) and the tetrasaccharide GlcN-β-1,4-GlcN-β-1,4-GlcN-β-1,4-GlcNAc_R_ (DDDA). We demonstrate that the use of this enzyme allows the production of oligomers as single glycoforms directly from heteropolymeric chitin or from crude lobster shells. We found *Pp*Chi to possess the best activity toward chitosan with a degree of acetylation (da) of 48%. The enzyme is highly stable, and multi-milligram amounts of homogenous oligomers have been prepared using a simple pre-pack carbon cartridge. Our results demonstrate the potential of using *Pp*Chi as a tool to produce paCOSs in a large scale for applications in the food, pharmaceutical, and agricultural sectors.

## Experimental Section

### Materials, Bacterial Strains, and Plasmids

*E. coli* competent cells and the pET-21b(+)vector were obtained from Thermo Fisher Scientific (Waltham, MA). Chitin from shrimp shells with a da of 90% was purchased from Sigma-Aldrich (St. Louis, MO), and chitosan with a da of 48% was a gift from Prof. Finn Aachmann (NTNU, Norway). Chitosan with a da of 10% was obtained from Mahtani Chitosan PVT Ltd (Gujarat, India). All other reagents were of analytical grade unless otherwise stated.

### Cloning of the *PpChi* Gene and Transformation of*E. coli*

The putative chitinase gene *BK*122*_*02780 from *P. pabuli* was codon-optimized for expression in *E. coli* and synthesized by GeneArt (Thermo Fisher Scientific, Waltham, MA) (Figure S1). The use of SignalP (www.cbs.dtu.dk/services/SignalP/) revealed that the *BK*122*_*02780 gene contains 96 bp that encode a predicted signal peptide at the *N*-terminal end of the corresponding protein. A template of the *BK*122*_*02780 gene not including the region coding for the predicted signal peptide was amplified by PCR using the Q5 HF polymerase master mix (New England Biolabs, MA), and the resulting products were cloned into the pET-21b(+) vector between the *NdeI* and *XhoI* restriction cloning sites using T4 DNA ligase (Thermo Fisher Scientific, MA). The sequences were verified at the EMBL sequencing facility (Heidelberg, Germany). The final constructs were transformed into One Shot BL21 *E. coli* competent cells (Thermo Fisher Scientific, MA) by heat shock at 42 °C for 45 s, before spreading and selecting transformants on ampicillin plates (Luria–Bertani broth (LB) medium containing 50 mg antibiotic per L).

### Heterologous Expression and Purification of the *Pp*Chi Protein

The selected *E. coli* cells carrying *BK*122*_*02780-pET-21b(+) were grown in LB medium supplemented with ampicillin (50 mg/L) at 37 °C on an orbital shaker (200 rpm) until the absorbance at 600 nm reached 0.6–0.8. Protein expression was induced by the addition of 0.5 mM isopropyl β-d-1-thiogalactopyranoside (IPTG) (Amresco, Solon, OH) at the optimized temperature of 16 °C. The cells were grown in these conditions at 180 rpm for further 18 h and harvested by centrifugation at 4000*g* for 15 min prior to lysis by ultrasonication. After centrifugation (16,000*g*, 1 h), the cell-free supernatants were collected and passed through a His-Trap column (GE Healthcare, Uppsala, Sweden), and the recombinant proteins were eluted using 20 mM sodium phosphate (pH 7.4) elution buffers containing 0.5 M NaCl and increasing imidazole concentrations (50, 100, 200, 300, and 1000 mM). The fractions were analyzed by sodium dodecyl sulfate polyacrylamide gel electrophoresis (SDS-PAGE), and those containing the target protein of approximately 55 kDa were collected and concentrated using an Amicon ultra-centrifugal filter unit (MW cut-off value of 10,000; Millipore, Cork, Ireland). Final protein concentration was determined using the Bradford dye-binding assay (Bio-Rad, Hercules, CA). The identity of the purified BK122_02780 (*Pp*Chi) protein was confirmed using tryptic peptide fingerprinting as described earlier.^34^

### Substrate Specificity

Substrate specificity was determined using chitin/chitosan with the da of 10, 48, and 90%. Chitohexaose (Megazyme, Wicklow, Ireland), Avicel (Sigma-Aldrich, St. Louis, MO), and 4-*O*-methyl glucuronoxylans (Sigma-Aldrich, St. Louis, MO) were also tested. The recombinant *Pp*Chi protein (0.2 nmol) was incubated with 1 mg of each substrate in 200 μL of 20 mM sodium acetate buffer (pH 6.0) for 20 min at 40 °C, and the reactions were subsequently stopped by boiling the mixtures for 5 min. The same experiments were also carried out over a 48 h incubation time. The resulting enzymatic reaction products were analyzed by matrix-assisted laser desorption ionization time-of-flight mass spectrometry (MALDI-ToF MS, Applied Biosystems, CA, USA) as described earlier.^34^

### 3-Methyl-2-Benzothiazolinone Hydrazone (MBTH) Assay

Chitin hydrolysis by *endo*-chitinases often results in a mixture of chito-oligosaccharides with various DPs. The MBTH reducing sugar assay was used to quantify chitin degradation as it is independent of oligosaccharide length.^35^ As described in the literature, the enzyme hydrolysates (100 μL) were mixed with 0.5 M NaOH (100 μL), to which equal volumes of freshly made 3 mg mL^–1^ MBTH and 1 mg/mL DTT were added. The reaction mixtures were heated for 15 min at 80 °C before a solution containing 0.5% (FeNH_4_(SO_4_)_2_)·12H_2_O, 0.5% sulfamic acid and 0.25 M HCl (200 μL) was added. The final mixtures were cooled to room temperature before absorbance was measured at 620 nm. All experiments were performed in triplicate.

### Optimal pH and Temperature

**Figure 1:**
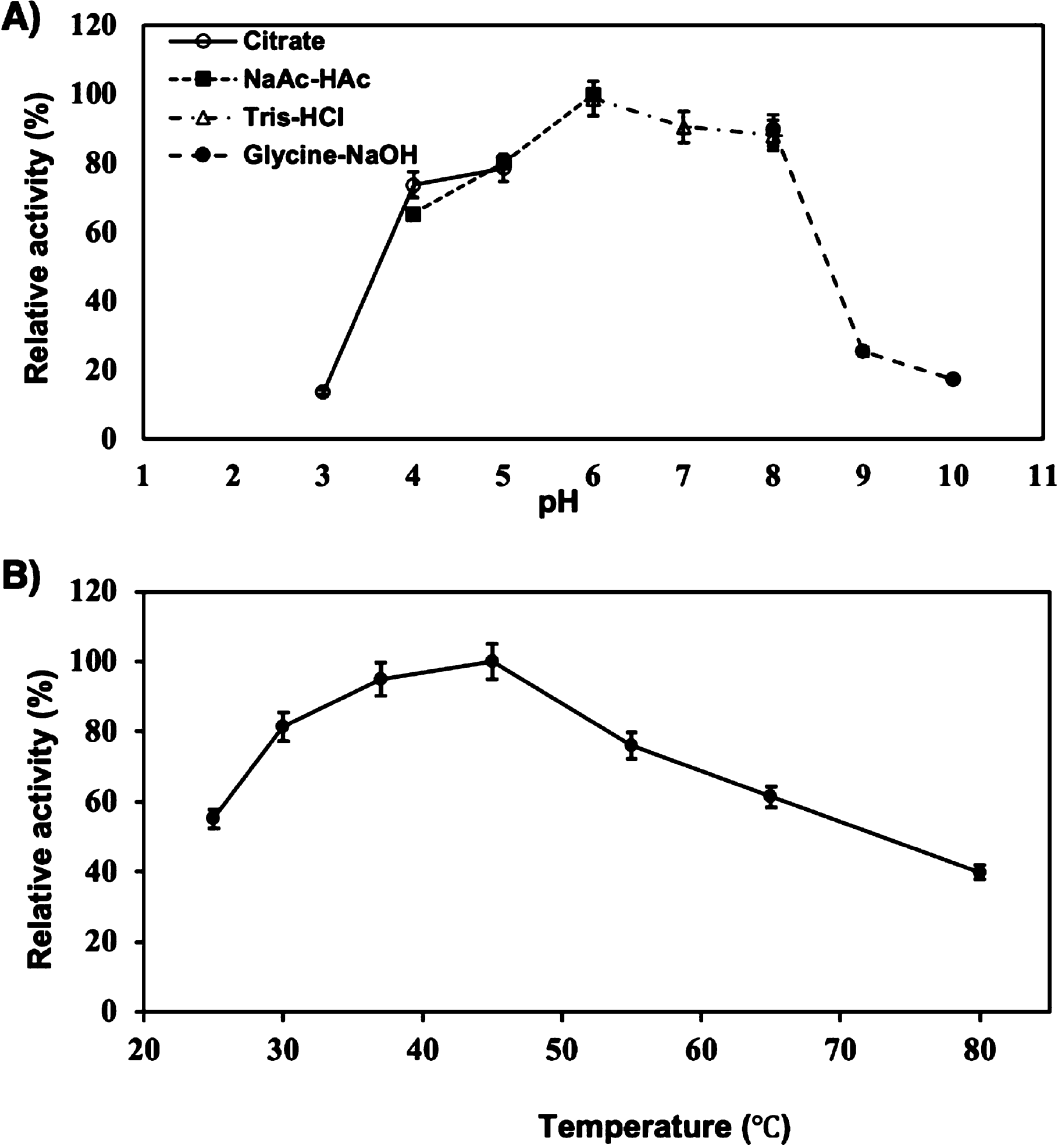
(A) Effects of pH on the activity of *Pp*Chi. Enzymatic reactions were performed using chitin with a da of 48% as a substrate and incubations were performed at 40 °C for 30 min at different pH values using various buffers, namely 20 mM sodium citrate (pH 3.0–5.0), sodium acetate (pH 4.0–6.0), Tris–HCl (pH 6.0–8.0), and glycine–NaOH (pH 8.0–10.0). B) Relative activity of *Pp*Chi at different temperatures was measured using chitosan with a da of 48% as a substrate at pH 6.0 for 30 min. Error bars indicate standard deviations of three experimental replicates.

To determine its optimum pH of action, the recombinant *Pp*Chi protein (0.2 nmol) was incubated at 40 °C for 1 h with chitosan of a da of 48% (400 μg) in 200 μL of the universal buffer (20 mM citrate buffer, 20 mM NaOAc Tris-HCI, and 20 mM Glycine-NaOH) adjusted to pH values in the range of 3 to 10 (Figure 1A). The optimal temperature of the enzyme was determined in the same conditions as mentioned above by incubating *Pp*Chi (0.2 nmol) at temperatures ranging from 25 to 80 °C (Figure 1B) for 30 min in 200 μL of 20 mM NaOAc buffer at pH 6.

### Characterization of *Pp*Chi Enzymatic Activity

To determine the activity of the recombinant *Pp*Chi protein, 2 nmol enzyme was incubated in 100 μL of 20 mM NaOAc buffer (pH 6.0) at 40 °C for 20 min in the presence of chitin or chitosan at concentrations ranging from 0.1 to 1 mg/mL under the optimal pH and temperature conditions. The enzymatic reaction was quenched by immersing the test tubes in boiling water for 5 min. Relative enzyme activity was measured by using the MBTH method, as described above. One unit of activity was defined as the quantity of enzyme required to release 1 μmol reducing sugar (based on a GlcNAc standard curve) per min in the above enzymatic reaction conditions. Kinetic parameters (*V*_max_, *K*_M_, and TN) were determined from Lineweaver–Burk plots of the reaction performed at different substrate concentrations.

### Oligosaccharide Purification and Structural Characterization

Chitosan with a da of 48% (30 mg) was mixed with the recombinant *Pp*Chi protein (15 nmol) and incubated at 40 °C for 1 h. The oligosaccharides were eluted separately from carbon cartridges using a 1–30% acetonitrile gradient. After drying in a centrifugal evaporator (SpeedVac, Thermo Fisher Scientific, Waltham, MA), the purified trisaccharide DDA and tetrasaccharide DDDA were weighed and analyzed by HPAEC-PAD (Table S1) and MALDI-TOF MS; MALDI CID MS/MS analysis was performed on a MALDI TOF/TOF 5800 system (AB Sciex, Framingham, MA).^34^

### Production of Chito-Oligosaccharides from Lobster Shells

Lobster shell powder was prepared as described earlier, using a modified procedure.^36^ A protease treatment (Alcalase from *Bacillus licheniformis*, Aldrich, St. Louis, MO; 1.5 U; 16 h, 25 °C) was carried out after an incubation in the presence of 25% NaOH containing 1% NaBH_4_ (10 mL; stirring for 16 h at ambient temperature). The material was dialyzed and freeze-dried, and the resulting shell preparation was divided and subjected to the following treatments, as described in ref (34): (1) *Pp*Chi (10 nmol) and shell preparation (20 mg) in 1 mM ascorbic acid, 20 mM NaOAc buffer (pH 6.0, 500 μL) at 40 °C, without *Ff*AA11 (fungal Auxilary Activity (AA11) oxidase); (2) *Pp*Chi (10 nmol) and shell preparation (20 mg) in 1 mM ascorbic acid, 20 mM NaOAc buffer (pH 6.0, 500 μL) at 40 °C, in the presence of 100 μM Cu^2+^-saturated *Ff*AA11; and (3) shell preparation with *Ff*AA11 (100 μM) incubated for 24 h, after which the insoluble pellet was collected by centrifugation, washed with water, dried, and treated (20 mg) with *Pp*Chi (10 nmol) at 40 °C for 24 h. All experiments were performed in triplicate.

## Results and Discussion

### Bioinformatic Analysis and Heterologous Expression of *Pp*Chi

The sequence of *BK*122*_*02780 (GenBank accession #OME85809.1) encoding a putative chitinase (*Pp*Chi) was identified by a BLAST search of the known chitinase catalytic domain. The gene sequence was codon-optimized for expression in *E. coli* and chemically synthesized (Figure S1). The corresponding protein sequence annotation suggests that *Pp*Chi is related to chitinases from the GH 18 family, although sequence identity with other identified GH 18 chitinases is low. For example, the closest biochemically characterized homologs are ChiA1 from *Bacillus circulans* and ChiA and ChiB from *Serratia marcescens*, to which *Pp*Chi shows sequence similarities of only 28, 22.6, and 26%, respectively (Figures S2, S3). *Pp*Chi also presents 25.8% sequence identity to chain B of another putative chitinase (ChiW) from *Paenibacillus* sp.^37^ Sequence alignment revealed the presence in *Pp*Chi of the conserved DXDXE motif and its characteristic catalytic aspartic and glutamic acids (Figure S4). To investigate the activity and specificity of *Pi*Chi, the protein was expressed in *E. coli* with a *C*-terminal His_6_ tag and purified to homogeneity by affinity chromatography. A total of 97 ± 14 mg of protein was produced per liter of LB broth, and the recombinant protein had an estimated molecular mass of 56 kDa on SDS-PAGE gels (Figure S5). The identity of the purified recombinant *Pi*Chi protein was verified by mass spectrometry, with a 79% sequence coverage (Figure S6).

### Substrate Specificity and Optimal Activity of the Recombinant *Pp*Chi

Despite little sequence similarities to known chitinases, our data show that the product of the *BK*122*_*02780 gene exhibits chitinolytic activity (Figure S7), but the enzyme did not hydrolyze cellulose and hemicellulosic substrates. Given its confirmed chitinolytic activity, we have named the BK122_02780 protein *Pp*Chi for “*P. pabuli* chitinase”. Interestingly, *Pp*Chi did not hydrolyze hexa-acetyl-chitohexaose, which led us to speculate that the protein has unusual subsites with a distinctive geometry toward chitin substrates.

The impact of pH and temperature on enzyme activity were evaluated by utilizing buffers with a pH range between 3.0 and 10.0 and by incubating the reaction mixtures at temperatures ranging from 25 to 80 °C. These *in vitro* assays confirmed that the highest activity is at pH 6.0 in 20 mM NaOAc buffer (Figure 1A), whereas buffering the reaction at pH 3.0 and 9.0 led to a significant loss of enzyme activity, with only 13.7 and 25.5% relative enzymatic activity retained, respectively. The optimal temperature of *Pp*Chi is 45 °C in 20 mM NaOAc buffer (pH 6.0) (Figure 1B). In addition, the enzyme retains 95 and 81% activity at 30 and 37 °C, respectively. Interestingly, after 30 min incubation at 80 °C, the enzyme retained approximately 40% of its chitinolytic activity, suggesting that it is thermostable at relatively high temperatures, similar to some chitinases isolated from thermophilic bacteria and fungi.^27^ Finally, no activity loss was observed when the enzyme was kept at ambient temperature for 72 h or 16 h at 45 °C.

### Kinetic Properties of the Recombinant *Pp*Chi Enzyme

Most known chitinases exhibit low to moderate catalytic activities against colloidal chitins (Table 1). Our kinetic studies of *Pp*Chi using three different chitin substrates with different degrees of *N*-acetylation showed that two of the substrates were not efficiently hydrolyzed by *Pp*Chi. In particular, *Pp*Chi activity on chitin (90% da) was characterized by a *V*_max_ of 0.0003 mM·s^–1^ and a turnover number (TN) of 0.023 s^–1^. Similar data were obtained using chitosan with 10% da: *V*_max_ of 0.0003 mM·s^–1^ and TN of 0.011 s^–1^ (Figure S9). We conclude that the enzyme has a low turnover rate when colloidal chitin is used as a substrate, akin to most chitinases reported in the literature. Yet, when the chitin substrate with 48% da was tested, the catalytic rate of *Pp*Chi increased remarkably compared to that of the other substrates, with a *V*_max_ of 0.97 mM·s^–1^, a TN of 67.17 s^–1^, and a *K*_M_ of 186.95 mM. Chitinolytic enzymes can degrade partially acetylated chitosan to a certain extent but at a much slower rate, except for a rare *Ralstonia* sp. ChiA, which is catalytically more efficient against partially *N*-acetylated chitosan than homopolymeric chitin or chitosan. We postulate that the *Ralstonia* sp. ChiA exhibits a specific subsite structure, allowing the binding of substrate segments composed of GlcN and GlcNAc residues in a specific pattern, and this could also be the case for *Pp*Chi. Based on our kinetics study, the turnover rate of *Pp*Chi on chitin with a da of 48% is 29 × 10^3^ times higher than that on homopolymeric chitin (90% da) and 61 × 10^3^ times faster than that on chitosan with 10% da.

**Table 1:** Bacterial Chitinases and Their Turnover Rate with Different Substrates as Reported in the Literature and Our Study

organisms	turnover (s^–1^)	substrate	ref
*Serratia marcescens*	1.7	beta chitin	(Hamre, Eide, Wold, & Sorlie, 2015)^39^
*Bacillus circulans*	9.55	carboxymethyl chitin	(Watanabe et al., 2003)^40^
*Thermococcus chitonophagus*	0.005	chitin	(Andronopoulou & Vorgias, 2003)^41^
*Vibrio harveyi*	1.2	colloidal chitin	(Pantoom, Songsiriritthigul, & Suginta, 2008)^42^
*Thermococcus chitonophagus*	0.0025	chitosan	(Andronopoulou & Vorgias, 2003)^41^
*Rhizomucor miehei*	0.009	colloidal chitin	(Yang, Fu, Yan, Jiang, & Wang, 2016)^43^
*Vibrio harveyi*	0.1	colloidal chitin	(Suginta, Pantoom, & Prinz, 2009)
*Penicillium ochrochloron*	2.37	colloidal chitin	(Patil, Waghmare, & Jadhav, 2013)^44^
*Scorpaena scrofa*	5.33	colloidal chitin	(Laribi-Habchi, Dziril, Badis, Mouhoub, & Mameri, 2012)^45^
*P. pabuli*	0.023	90% da chitin	this study
*P. pabuli*	0.011	10% da chitosan	this study
*P. pabuli*	67.17	48% da chitosan	this study

### Purification and Structural Characterization of the Chito-Oligosaccharides Released by the Recombinant *Pp*Chi Protein

**Figure 2:**
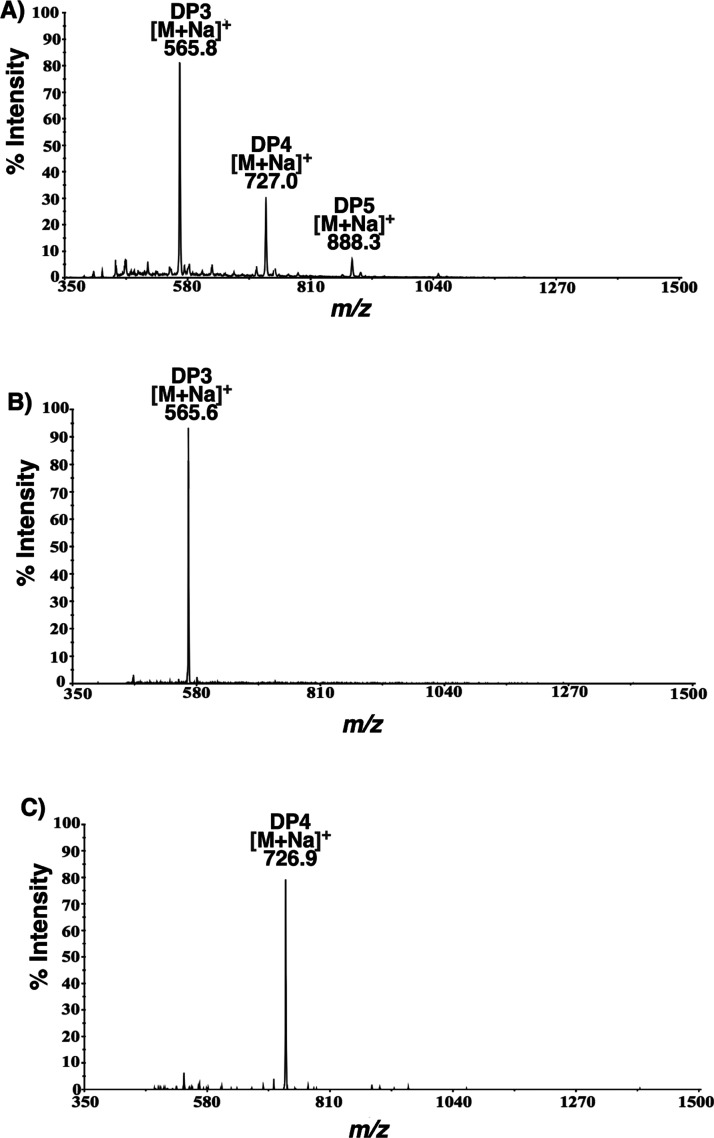
Mass spectra of (A) products released by *Pp*Chi incubated in the presence of chitosan with a da of 48% and (B,C) purified GlcN_2_GlcNAc_1_ (DP3) and GlcN_3_GlcNAc_1_ (DP4) oligomers.

The molecular weights of the chitin oligosaccharides formed by the recombinant *Pp*Chi were determined by mass spectrometry, with compositional information derived from MS1 spectra. Unlike the commercially available chitinase tested here, which liberate heterogeneous oligomers, for example, sodiated molecular ion [M + Na]^+^ of GlcNAc_2_GlcN_1_*m/z* = 608; GlcNAc_3_*m/z* = 650; GlcNAc_3_GlcN_1_*m/z* = 811; GlcNAc_4_*m/z* = 853; GlcNAc_4_GlcN_1_*m/z* = 1015; and GlcNAc_5_*m/z* = 1057, from chitins with 48% da (Figure S7A), hydrolysis by *Pp*Chi led to a simpler oligosaccharide profile consisting of oligomers with *m/z* values of 566, 727, and 888. These correspond to [M + Na]^+^ of GlcN_2_GlcNAc_1_ (DP3), GlcN_3_GlcNAc_1_ (DP4), and GlcN_4_GlcNAc_1_ (DP5), respectively (Figure 2A).

**Figure 3:**
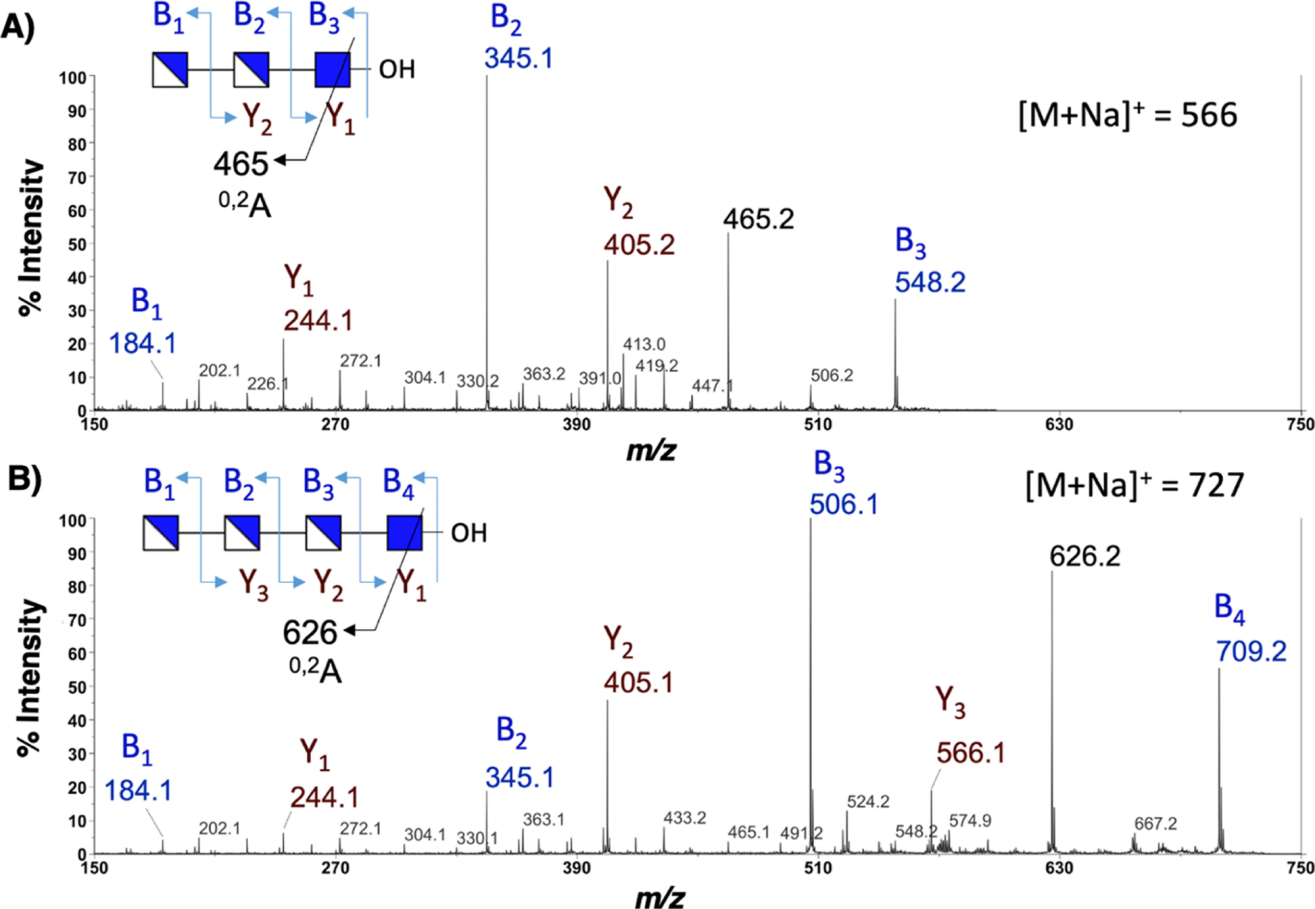
MALDI TOF/TOF MS/MS spectra of paCOSs. (A) GlcN-β-1,4-GlcN-β-1,4-GlcNAc_R_ (DDA) and (B) GlcN-β-1,4-GlcN-β-1,4-GlcN-β-1,4-GlcNAc_R_ (DDDA).

The *Pp*Chi hydrolysates of two homopolymeric substrates also contained identical GlcNAcGlcN_(*n*)_, with *n* = 2–4 (Figure S7), suggesting that *Pp*Chi possesses distinct subsite specificity accommodating GlcN and GlcNAc residues in a specific arrangement. To confirm this, we isolated the oligosaccharides produced from 30 mg chitosan with a da of 48% (30 mg) upon treatment with 15 nmol *Pp*Chi, using carbon SPE cartridges. The two most abundant oligomers, that is, the DP3 (*m/z* 566) and DP4 (*m/z* 727) compounds, were purified to homogeneity (Figures 2B,C, S8), with yields of 10.2 ± 1.7 and 1.9 ± 0.5 mg, respectively. The isolated yield was higher than expected and could be a result of the presence of contaminant salt. The chemical structure of each oligomer was analyzed separately using MS2. The sodiated molecular ion of the DP3 oligosaccharide produced a diagnostic pair of sodiated B_2_ and Y_2_ ions at *m/z* 345 (GlcN-GlcNN-) and 405 (-GlcN-GlcNAc), respectively, thus locating the single GlcNAc at the reducing end of the glycan chain. This is further supported by other B and Y ions, as shown in Figure 3A. For the DP4 oligosaccharide, the sodiated B_2_ and B_3_ ions at *m/z* 345 and 506 also place the single GlcNAc at the reducing end of the molecule (Figure 3B). This is further corroborated by the sodiated Y_1_ ion at *m*/*z* 244, corresponding to a reducing end GlcNAc found in both DP3 and DP4 oligomers.

Our data therefore indicate that the two major isolated products are DDA and DDDA (Scheme 2A), although we could not rule out the presence of additional minor isomeric oligosaccharides. The subsite specificity of *Pp*Chi at −2, −3, and −4 positions and the requirement of a GlcNAc residue in the −1 subsite have contributed to the production of the DDA and DDDA oligomers (Scheme 2B). DDA and DDDA were also found in low abundance in the *Pp*Chi hydrolysates of two homopolymeric substrates (90% and 10% acetylation). Their formation in small amounts may be the result of limited substrate binding sites, thus failing to provide enough necessary contact points along the glycan chains for *Pp*Chi to exert its catalytic activity.

To investigate the activity further, we have carried out extensive 48 h incubations of *Pp*Chi with all three substrates. These experiments revealed that, in addition to the previously identified DDA (*m/z* 566), DA (*m/z* 405) and AA (*m/z* 447) were also detected in the hydrolysates of two chitin substrates (da 90% and da 48%) (Figure S10). When the same treatment was applied to chitosan (da 10%), only DA (*m/z* 405) and DDA (*m/z* 566) were detected by MS (Figure S10). These data further confirm that the production of DDA is the highest, but longer enzymatic hydrolysis allow the production of shorter oligomers.

### Production of DDA and DDDA Oligosaccharides from Lobster Shells Assisted by a Lytic Polysaccharide Monooxygenase

The use of chitinases in marine biorefinery processes is often accompanied by low yields, especially when dealing with crystalline substrates and heterogenous crustacean biomass. The auxiliary activity 11 (AA11) enzymes are a recently discovered fungal chitin-specific lytic polysaccharide monooxygenases (LPMO) that have the ability to enhance the breakdown of resilient chitin substrates.^34,46−48^ To further demonstrate the potential to exploit *Pp*Chi for the production of chito-oligosaccharides from raw biomass, we have optimized the preparation of DDA and DDDA from lobster shells by combining the activity of *Pp*Chi with the oxidizing power of a fungal LPMO, which in nature is responsible for assisting the degradation of recalcitrant biomass. In a previous report, we demonstrated that chitin breakdown from the lobster shell improves significantly when the activity of a *Fusarium fujikuroi* LPMO (*Ff*AA11) is combined with the action of a commercial chitinase (*Tv*Chi).^34^ In the present study, we first submerged the lobster shells in strong alkali to allow partial deacetylation of chitin and used *Pp*Chi together with *Ff*AA11 to release high amounts of DA, DDA, and DDDA (Figure S11). Our data show that this two-step approach leads to a sixfold increase in the production of the chito-oligomers compared to that with the treatment with *Pp*Chi only. In addition, a 1.4-fold increase is observed when both *Ff*AA11 and *Pp*Chi are combined in a one-pot reaction, suggesting that the two enzymes do not work synergistically (Table S2). Thus, the two-step biocatalytic approach combining the oxidative power of the LPMO and hydrolytic activity of *Pp*Chi represents a potential platform for the treatment of marine biomass and the production of chito-oligosaccharides with a defined structure that could be potentially exploited for the green and safe production of paCOSs for applications in food additives or food packaging materials.

In summary, a newly discovered bacterial chitinase, *Pp*Chi, has been characterized. The enzyme has low sequence similarity with other chitinases, and analysis of its amino acid sequence suggests that it contains a discrete GH-18 domain with Asp111 and Glu115 as essential catalytic amino acids. To the best of our knowledge, this is the first chitinase reported in the literature that releases predominately DDA and DDDA oligosaccharides from the partially de-acetylated chitin. When coupled with the action of *Ff*AA11, the production of these oligomers from the lobster shell by *Pp*Chi is significantly enhanced. We postulate that chitinases with novel specificities, such as *Pp*Chi, will provide new means for the green production of structurally defined oligosaccharides akin to organic synthesis.

## Supporting Information Available

*Pp*Chi codon-optimized nucleotide sequence; sequence alignment of *Pp*Chi and other characterized chitinases; phylogenic trees of bacterial chitinases that were biochemically characterized; catalytic domain alignment of *Pp*Chi and other fully characterized chitinases; SDS-PAGE analysis of the purified recombinant *Pp*Chi protein using a Ni-NTA column; peptides identified by mass spectroscopic analysis of the recombinant *Pp*Chi protein; mass spectra of products released by *Tv*Chi incubated for 1 h in the presence of chitosan with a da of 48% and products released by *Pp*Chi after 1 h treatment with chitin (90% da) and chitosan (10% da); HPAEC-PAD chromatogram of purified DP3 (DDA) and DP4 (DDDA); Lineweaver–Burk plots of *Pp*Chi in the presence of chitin with a da of 90, 48, and 10%; mass spectra of products released by *Pp*Chi after 48 h incubation in the presence of chitin with a da of 90, 48, and 10%; mass spectrum of chito-oligosaccharide products released by *Pp*Chi after 24 h treatment of the lobster shell preparation pretreated with *Ff*AA11; gradients and eluents of HPAEC-PAD; and hydrolysis of lobster shell samples in different conditions (PDF)
