# Supplementary material for: Production of Structurally Defined Chito-Oligosaccharides with a Single N-Acetylation at Their Reducing End Using a Newly Discovered Chitinase from Paenibacillus pabuli
Source: J Agric Food Chem. 2021 Mar 10;69(11):3371–9. doi: 10.1021/acs.jafc.0c06804 (PMC8041281; doi:10.1021/acs.jafc.0c06804)
Supplement: Supplementary file 1 — jf0c06804_si_001.pdf [file jf0c06804_si_001.pdf]

Supporting Information

Production of structurally defined chito-oligosaccharides with a single *N*-acetylation at their reducing end using a newly discovered chitinase from *Paenibacillus pabuli*

Jing Li[a,b,c], Damao Wang[b,d], Shu-Chieh Chang[b], Pi-Hui Liang[e], Vaibhav Srivastava[b], Shih-Yun Guu[f], Jiun-Jie Shie[g], Kay-Hooi Khoo[f], Vincent Bulone[b,h], Yves S. Y. Hsieh*[b,c,i]

[a]College of Life Sciences, Shanghai Normal University, Shanghai 220234, PR China

[b]Division of Glycoscience, Department of Chemistry, School of Engineering Sciences in Chemistry, Biotechnology and Health, Royal Institute of Technology (KTH), AlbaNova University Center, Stockholm, SE10691, Sweden

[c]School of Pharmacy, College of Pharmacy, Taipei Medical University, 250 Wuxing Street, Taipei 11031, Taiwan

[d]College of Food Science, Southwest University, Chongqing, 400715, PR China

[e]School of Pharmacy, College of Medicine, National Taiwan University, Taipei 100, Taiwan

[f]Institute of Biological Chemistry, Academia Sinica, 128 Academia Road Sec. 2, Nankang, Taipei 115, Taiwan

[g]Institute of Chemistry, Academia Sinica, 128 Academia Road Sec. 2, Nankang, Taipei 115, Taiwan

[h]School of Agriculture, Food and Wine, The University of Adelaide, Urrbrae 5064, Australia

[i]Genomics Research Center, Academia Sinica, 128 Academia Road Sec. 2, Nankang, Taipei 115, Taiwan

GGAAGTGCTCCACTACCCAAGAAAATTATAGCGTATGTTGCGGGTTGGGCAAACTGGACC
GCTAACGACATCAAAGCCGAACAGCTCAGCCACATTAATTATTCGTTCGCCTTAATCAGC
AATGGCAAGGCGACTATCACCAACAGCGACCGCACCAAACTGCAAATGATGGTTGGCCTG
AAGAGCCGTAATCCGGACCTGAAAGTGCTGCTCTCTGTGGGCGGTTGGGGTGCGAACGGC
TTTAGCGACGCAGCGCTGACAGATGCGTCCCGCACGACGTTTGCAGATAGCATTGTCCAG
TTGGTGACCTCGAATAACCTGGACGGCGTGGACCTCGACTGGGAGTACCCGACGAACCCG
GCAGCTGGCACCACCGCGCGTCCGCAAGATAAACAGAATTTTACCCAGCTGCTGTCGAAG
GTACGTGAAAAGTTGAACGCGCAAGGTCAGATCAACGGGAAACAATACCTGCTGACCATT
GCAGCGGGCGCTAGCTCCTCCTACCTGAACGGCGTGGAAATTAACAACATTACCCCGCTG
TTGGACTGGATTAACCTGATGACCTACGACTTCCACGGCACCTGGGATGCCACCACGGGT
CATCATACCAATCTGAGCGGTCGCGATATCAGCGTCACCTCCGCTGTGAACCTGTTCCGC
AACTCAGGCGTTCCGGCGAATAAGCTAGTGATTGGTGGTGCCTTCTATGGTCGTGCGTGG
ACCGGCGTTCAGAACTCTAATAACGGCCTGGACCGTCCGGGATCTGGTGGTTTCGAACCG
GACTACAACACCATCGTTTCTCAGTACCTCAACAAGAACGGTTATACTCGTTACTGGGAC
AGCTCCGCACAAGCTCCGTATTTGTTCAACGGCAATACCTTTATCTCTTACGATGATCCG
CAGTCTCTGAGCCTGAAGGTGCAATACGTTAAGAACAGCAACCTTGGTGGTATTATGTTT
TGGGAGTATTCCAACGACCGTTCCGGTGCATTGCTGCAAGCGGTCTATTCGGAAGTTACC
GGCGGCGGAACGGTTCAGCCTCCGAATCCAAGCGGTTACAACTATTTGGTAGCGCAGGCG
AATCAGCAGATCGTGAGCGCGGATAATCAAGGTAATGATCAGCTGGTGGCGAACCGTACC
ACGGCCGGTGATTGGGAGCTGTTCGAGTGGATCACCAACAGCGACGGTACGGTTTCCTTG
AAAAGCAAAATCAACAACAAATACGTCACTGCGGATGTGAATCTGGGTGGTGCTCTGATC
GCTAAAGCCACCACCATCCAGCAATGGGAGAAATTCAATCGTGTTGACCTGGGCGATGGC
ACTATTGCCCTGCAAGCGTTAGCAAACAACCTTTATGTTACGTGCGATTTGAATAATGGC
GGCAAGTTGGTCGCGAGCAGAAACAGCGTTGGTGGCGCTTGGGAGGCATTTCGCGTGAAC
AAGCTGGAA

Figure S1: *Pp*Chi codon-optimized nucleotide sequence

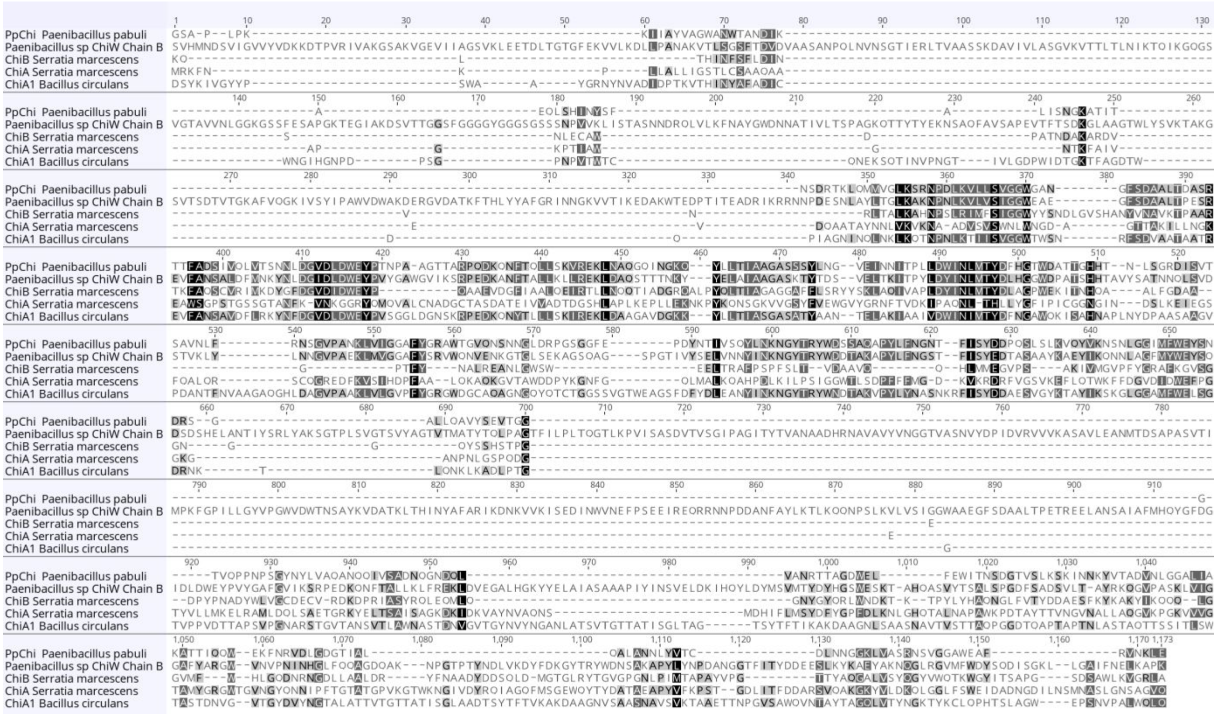

Figure S2: Sequence alignment of *Pp*Chi and other characterized chitinases

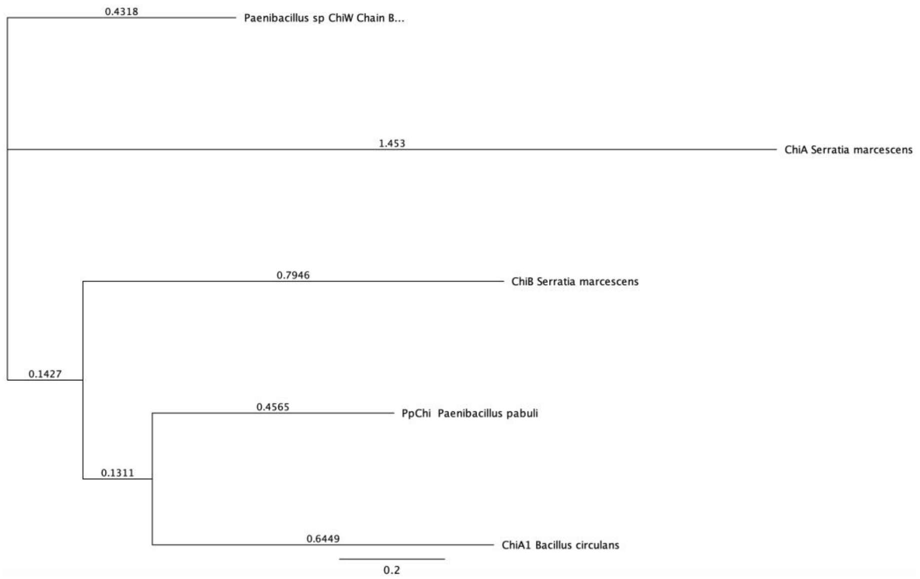

Figure S3: Phylogenetic trees of bacterial chitinases that were biochemically characterized.

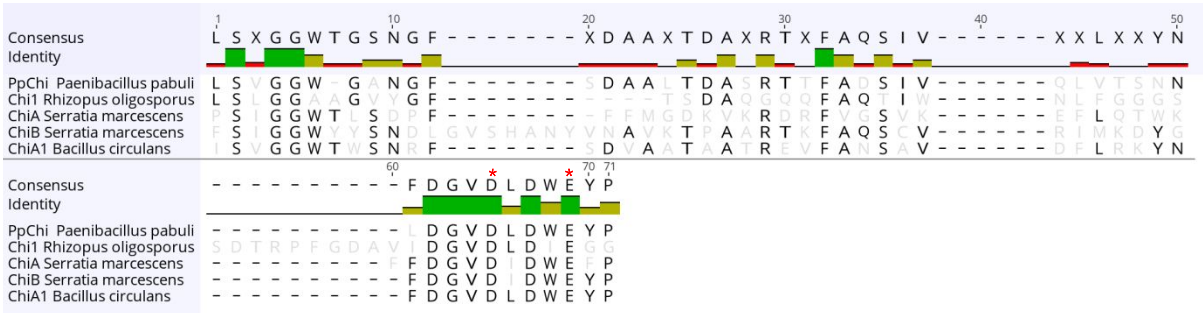

Figure S4: Catalytic domain alignment of *Pp*Chi and other fully characterized chitinases (catalytic amino acids are marked with red asterisks).

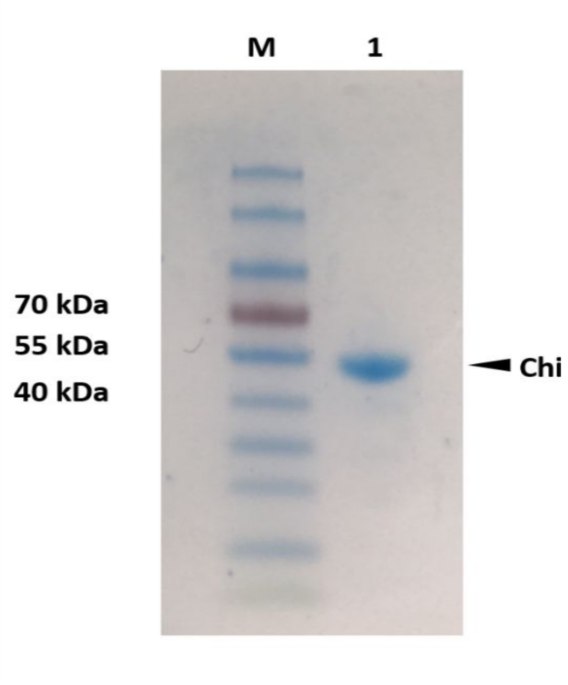

Figure S5: SDS-PAGE analysis of the purified recombinant *Pp*Chi protein: Lane M, protein markers; Lane 1, purified *Pp*Chi protein using a Ni-NTA column.

Protein sequence coverage: 79%

GSAPLPKKIIAYVAGWANWTANDIKAEQLSHINYSFALISNGKATITNSDRTKLQLMV
GLKSRNPDLKVILSVGGWGANGFSDAALTDASRTTFADSIVQLVTSNNLDGVDLDWE
YPTNPAAGTTARPQDKQNFTQLLSKVREKLNAQGQMNGKQYLLTIAAGASSSYLNGV
EINNITPLLDWINLMTYDFHGTWDATTGHHTNLSGRDISVTSAVNLFRNSGVPANKLV
IGGAFYGRAWTGVQNSNNGLDRPGSGGFEPDYNTIVSQYLNKNGYTRYWDSSAQAP
YLFNGNTFISYDDPQSLSLKVQYVKNSNLGGIMFWEYSNDRSGALLQAVYSEVTGGG
TVQPPNPSGYNYLVAQANQQIVSAENQGNDQLVANRTTAGDWELFEWITNSDGTVSL
KSKINNKYVTADVNVGGALIAKATTIQQWEKFNRVDLGDGTIALQALANNLYVTCDL
NNGGKLVASRNSVGGAWEAFRVNKLE

Figure S6: Peptides identified by mass spectrometric analysis of the recombinant *Pp*Chi protein (red).

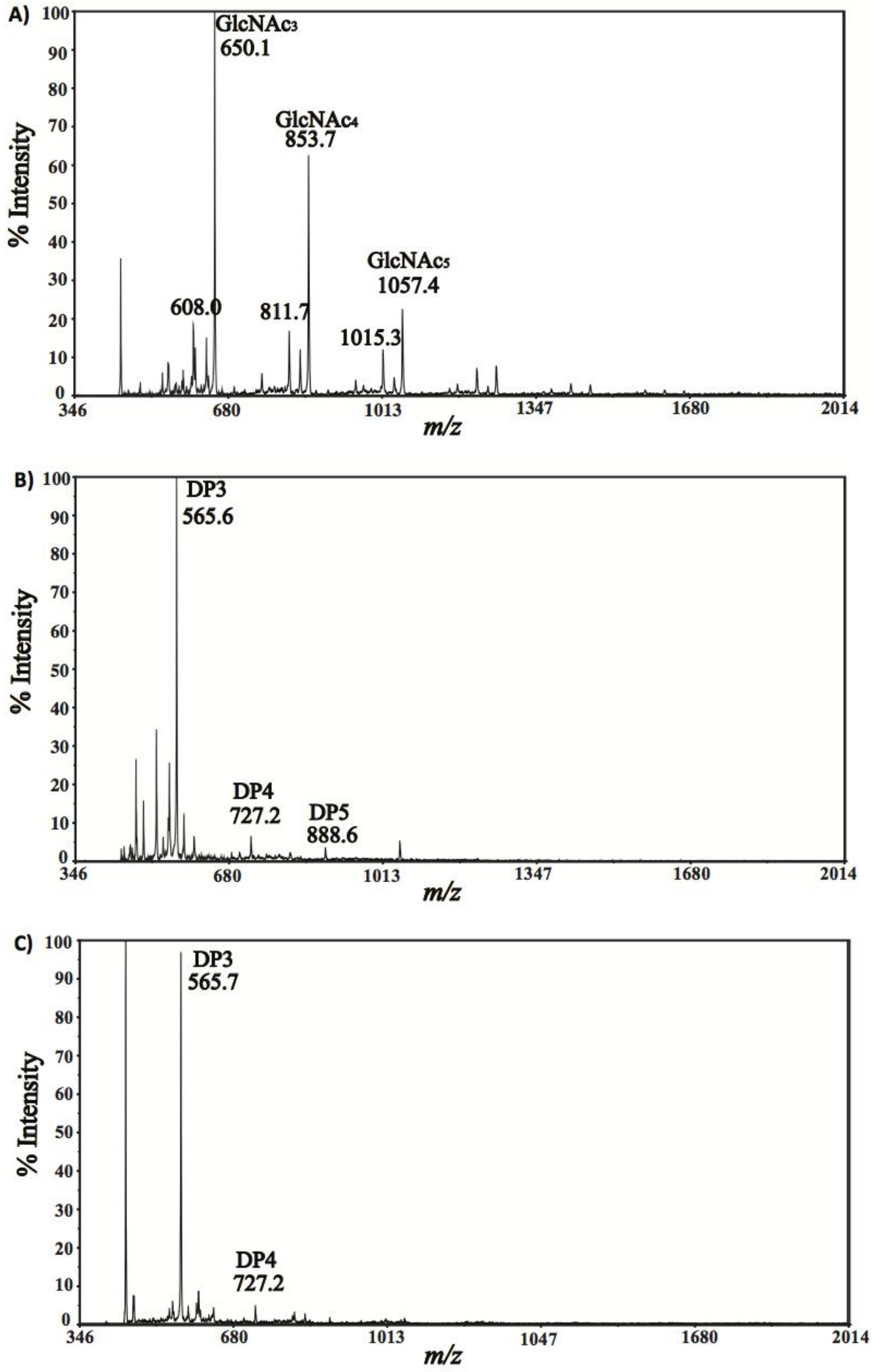

Figure S7: Mass spectra of: A) products released by *Tv*Chi incubated for 1 h in the presence of chitosan with a da of 48%; and B) and C), products released by *Pp*Chi after 1-h treatment with chitin (90% da) and chitosan (10% da), respectively

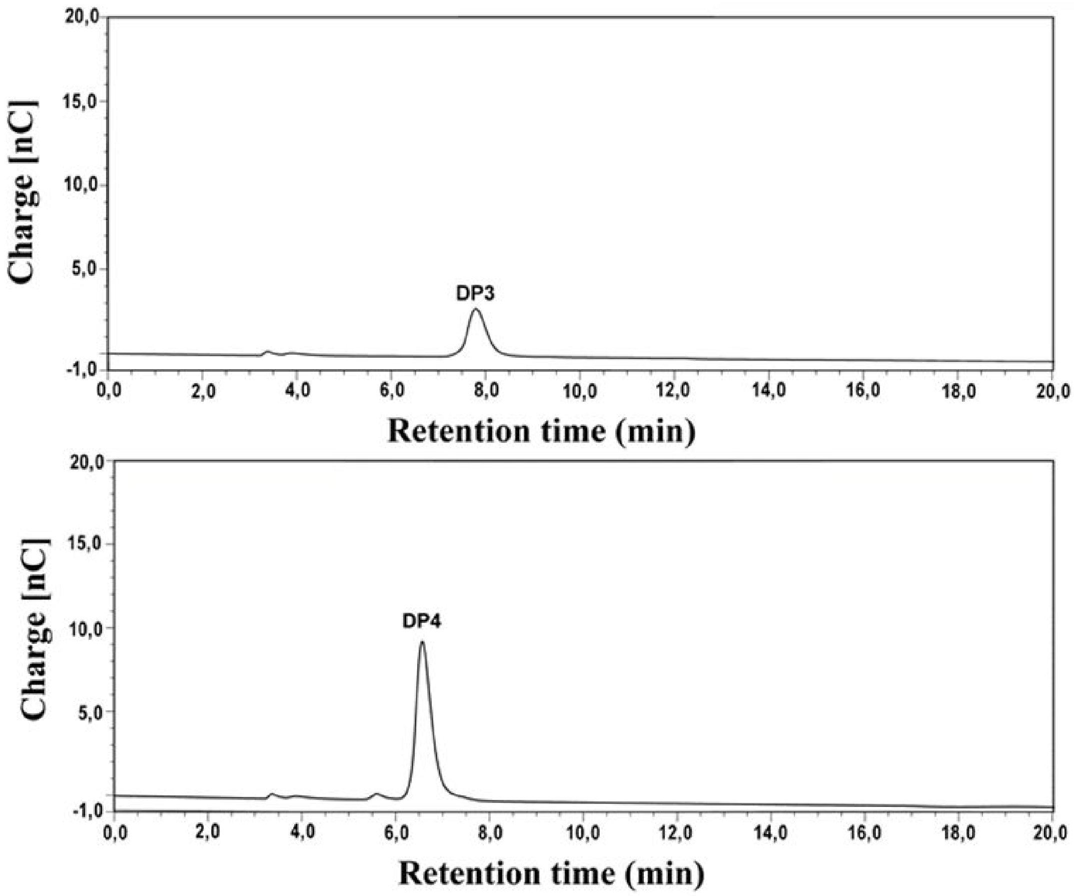

Figure S8: HPAEC-PAD chromatogram of purified DP3 (DDA) and DP4 (DDDA).

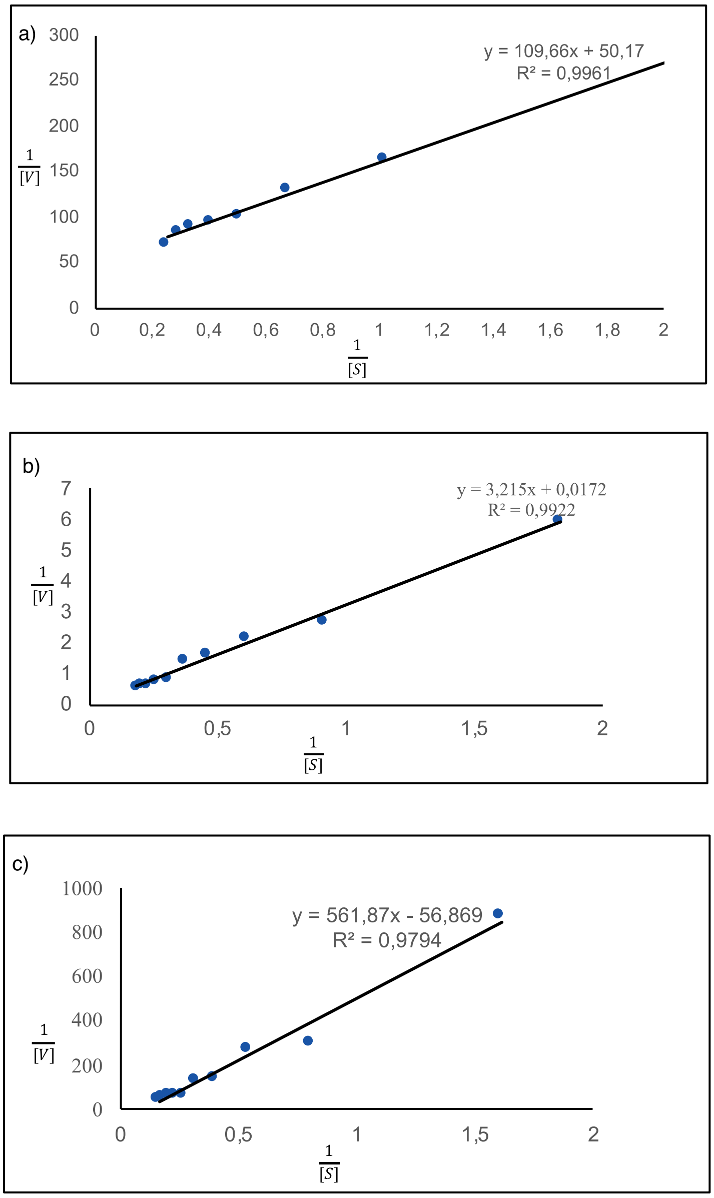

Figure S9: Lineweaver–Burk plots of *Pp*Chi in the presence of chitin with a da of A) 90%, B) 48%, and C) 10%.

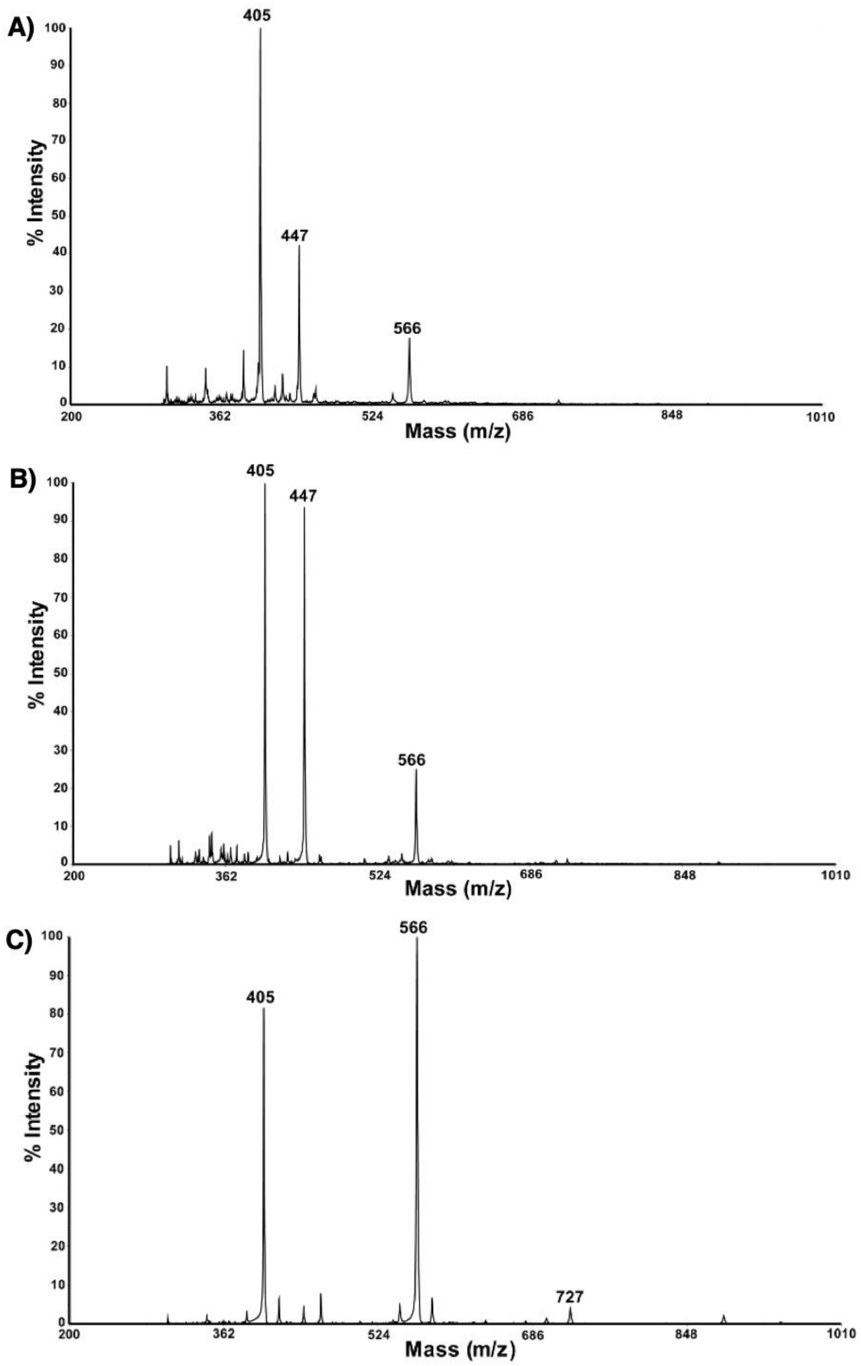

Figure S10: Mass spectra of products released by *Pp*Chi after 48h incubation in the presence of chitin with a da of A) 90%, B) 48%, and C) 10%. Sodiated molecular ion $[M+Na]^+$ of GlcNGlcNAc *m/z* = 405; GlcNAcGlcNAc *m/z* = 447; GlcN$_2$GlcNAc *m/z* = 566; GlcN$_3$GlcNAc *m/z* = 727.

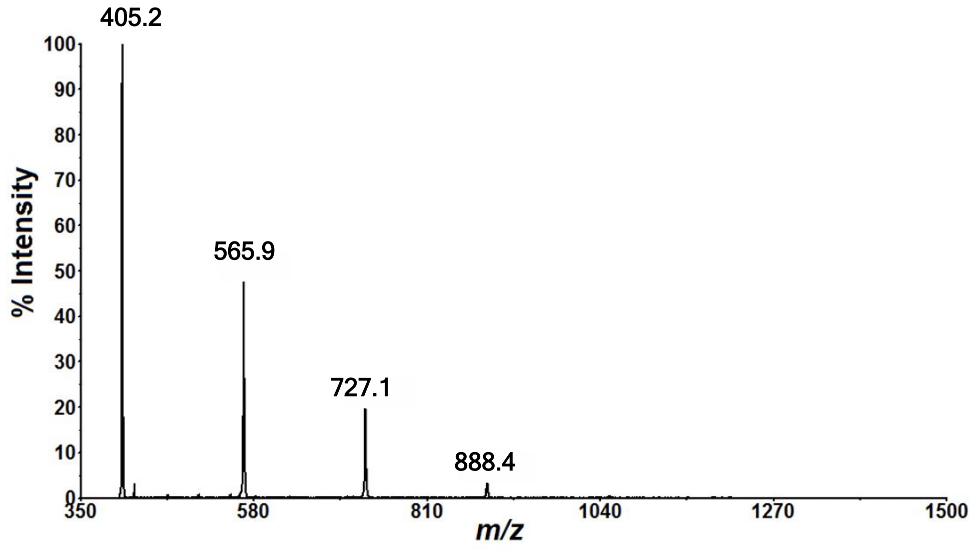

Figure S11: Mass spectrum of chito-oligosaccharide products released by *Pp*Chi after 24 h treatment of the lobster shell preparation pretreated with *Ff*AA11.

Table S1 Gradients and eluents of HPAEC-PAD (Dionex, Sunnyvale, CA)

Column: PA 200
Flow rate: 0.5ml/min
Injection volumn: 10 uL
Column temperature: 30 ˚C

Retention time (min)	300mM NaOH	1 M NaOAc	H_2O
0	7%	0%	93%
25	7%	0%	93%
30	7%	50%	43%

Table S2 Hydrolysis of lobster shell samples in different conditions

Time (h)	Reducing sugar (μM)		
	Lobster shell+*Pp*Chi	Lobster shell +*Ff*AA11 + *Pp*Chi	*Ff*AA11 pretreated lobster shell+ *Pp*Chi
2	1.00±0.17	1.14±0.09	6.19±1.59
4	2.88±0.45	4.51±0.84	18.24±3.80
8	7.85±0.91	10.41±2.07	43.01±8.25
24	9.39±1.18	17.07±3.94	63.85±7.48
48	16.38±2.30	24.40±4.87	104.68±13.59
72	17.79±3.51	24.10±4.16	110.24±10.96
